# Supplementary material for: Bipolar electrochemical tweezers using pristine carbon fibers with intrinsically asymmetric features
Source: Nat Commun. 2025 Nov 17;16:10061. doi: 10.1038/s41467-025-65036-z (PMC12624083; doi:10.1038/s41467-025-65036-z)
Supplement: Supplementary file 1 — Supplementary Information [file 41467_2025_65036_MOESM1_ESM.pdf]

Bipolar electrochemical tweezers using pristine carbon fibers with intrinsically asymmetric features

Bhavana Gupta[1,2], Vishal Shrivastav[1], Shashank Sundriyal[3], Ambrose Ashwin Melvin[4], Marcin Holdynski[1], Alexander Kuhn[5], Wojciech Nogala[1]**

[1]Institute of Physical Chemistry, Polish Academy of Sciences, Kasprzaka 44/52, 01-224 Warsaw, Poland

[2]Department of Chemistry, Cluster of Applied Sciences, School of Advanced Engineering, UPES, Uttarakhand, India

[3]Regional Center of Advanced Technologies and Materials, Czech Advanced Technology and Research Institute (CATRIN), Palacký University, Olomouc, Šlechtitelů 27, Olomouc 779 00, Czech Republic

[4]Department of Chemical & Biomolecular Engineering, Sogang University, 35 Baekbeom-ro, Mapo-gu, Seoul, 04107 Republic of Korea

[5]Univ. Bordeaux, CNRS, Bordeaux INP, ISM UMR 5255, 33607 Pessac, France

E-mail: wnogala@ichf.edu.pl,

E-mail: bhavana.gupta@ddn.upes.ac.in

Supplementary Video 1.

The electrochemistry of carbon fiber compared with human hair (10 x accelerated).

Supplementary Video 2.

Electrochemistry of carbon fiber with a rough surface downward motion (10 x accelerated).

Supplementary Video 3.

Electrochemistry of carbon fiber with a rough surface upwards motion (10 x accelerated).

Supplementary Video 4.

Up and down consecutive movement in carbon fiber (10 x accelerated).

Supplementary Video 5.

Dual carbon fiber-based electrochemical tweezer (10 x accelerated).

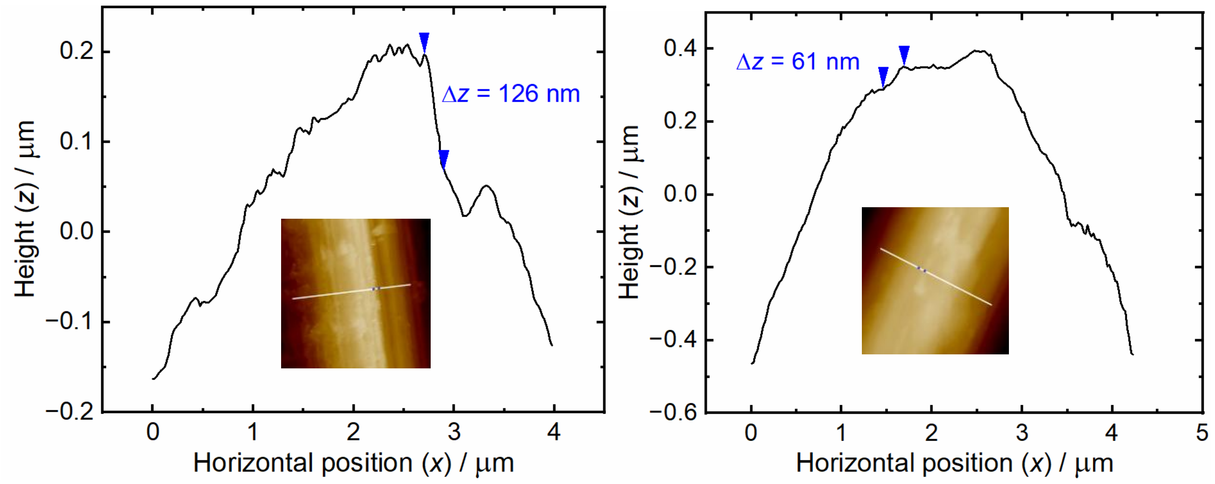

Supplementary Figure 1.

Cross-section profiles from AFM images. Profiles are extracted from AFM images in Fig. 1 i) (left plot) and j) (right plot). Positions of cross-section lines are shown in insets.

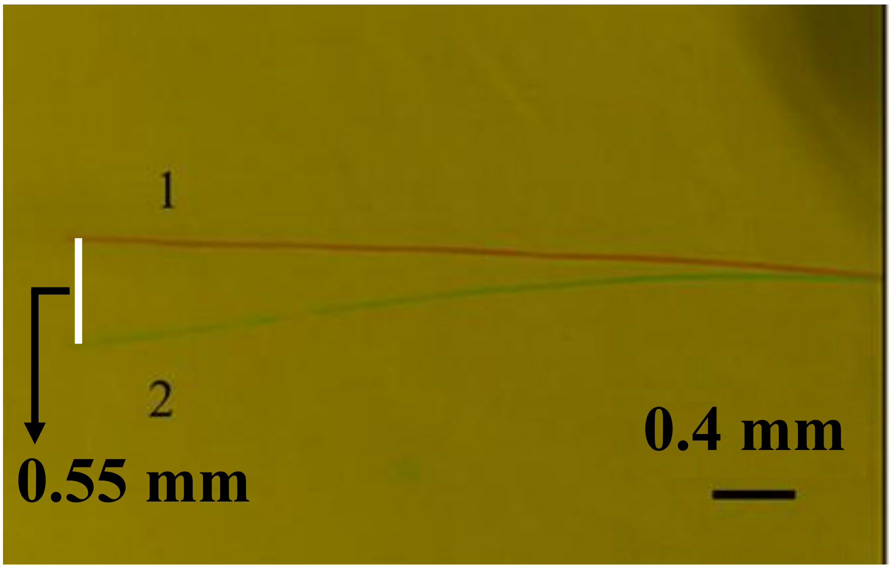

Supplementary Figure 2.

Carbon fiber actuation under 2 V and 3 V polarization. Overlayed micrographs of actuation in carbon fiber. 1) polarized at 2 V and 2) at 3 V.

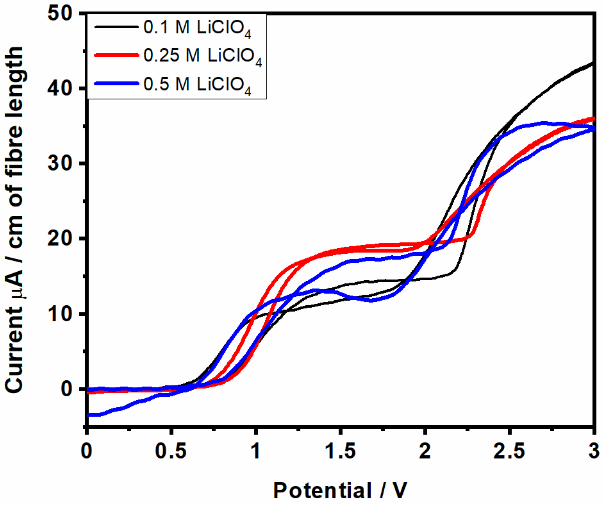

Supplementary Figure 3.

Effect of $LiClO_4$ concentration on cyclic voltammograms of carbon fiber CVs. The voltammograms are for concentrations of LiClO4: 0.1 mol dm^{-3} (black), 0.25 mol dm^{-3} (red), and 0.5 mol dm^{-3} (blue).

Supplementary Table 1. Deconvoluted peak energies in XPS spectra from Figure 5.

Element	Pristine CF	Treated CF	Bonding
	Bindig energy [eV]		
C 1*s*	-	283.1	carbides
	283.9	284.0	C=C
	284.8	284.8	C-C
	285.5	285.6	C-C/C-H
	286.2	286.7	C-O
	286.9		
	-	287.9	C=O
	-	289.0	O-C=O
O 1*s*	-	531.2	C=O
	532.4	532.4	C-O
Cl 2$p_{3/2}$	-	198.2	-
	-	199.8	C-Cl
