# Supplementary material for: Bipolar electrochemical tweezers using pristine carbon fibers with intrinsically asymmetric features
Source: Nat Commun. 2025 Nov 17;16:10061. doi: 10.1038/s41467-025-65036-z (PMC12624083; doi:10.1038/s41467-025-65036-z)
Supplement: Supplementary file 2 — Description of Additional Supplementary Files [file 41467_2025_65036_MOESM2_ESM.pdf]

Description of Additional Supplementary Files

File Name: Supplementary Video 1

Description: The electrochemistry of carbon fiber compared with human hair (10 x accelerated).

File Name: Supplementary Video 2

Description: Electrochemistry of carbon fiber with a rough surface downward motion (10 x accelerated).

File Name: Supplementary Video 3

Description: Electrochemistry of carbon fiber with a rough surface upwards motion (10 x accelerated).

File Name: Supplementary Video 4

Description: Up and down consecutive movement in carbon fiber (10 x accelerated).

File Name: Supplementary Video 5

Description: Dual carbon fiber-based electrochemical tweezer (10 x accelerated).
